# A national survey of the infectious diseases and antimicrobial stewardship pharmacist workforce in the United States: work settings, characteristics, employment activities, resources and needs

**DOI:** 10.1017/ash.2026.10312

**Published:** 2026-03-27

**Authors:** Elizabeth B. Hirsch, David A. Mott, Aaron M. Gilson, Samuel L. Aitken, Vicki Basalyga, Scott J. Bergman, Whitney R. Buckel, Kathryn E. DeSear, Margo Farber, Caroline A. Gaither, Alan E. Gross, Mary S. Hayney, Erin K. McCreary, Melanie R. Nicol, Warren E. Rose, Jon C. Schommer, Jamie L. Wagner, Tracy N. Zembles, Melissa D. Johnson

**Affiliations:** 1 University of Minnesota College of Pharmacy: University of Minnesota Twin Cities, USA; 2 University of Wisconsin-Madison School of Pharmacy, USA; 3 University of Michigan Department of Pharmacy, USA; 4 ASHP: American Society of Health-System Pharmacists, USA; 5 Nebraska Medicine-Nebraska Medical Center: Nebraska Medicine, USA; 6 Intermountain Health, USA; 7 University of Florida Department of Pharmacy Education and Practice, USA; 8 Wayne State University, USA; 9 University of Illinois Chicago, USA; 10 University of Pittsburgh Department of Infectious Diseases, USA; 11 Mount Auburn Hospital, USA; 12 Childrens Hospital of Wisconsin: Milwaukee Hospital-Children’s Wisconsin, USA; 13 Duke University Medical Center: Duke University Hospital, USA

## Abstract

**Purpose:**

This national survey aimed to describe the work settings, characteristics, employment activities, scope, functions, and challenges of the pharmacist workforce responsible for infectious diseases (ID) and antimicrobial stewardship (AMS)-related tasks in the U.S.

**Methods:**

An internet-based *Qualtrics* ^*XM*^ survey was distributed to 22,749 unique individuals with potential ID or AMS job responsibilities via email listservs for three national pharmacy organizations, and was open from 9/25/2024 to 10/24/2024. A respondent was considered engaged in ID/AMS activities if they reported involvement in at least one of 14 activities directly related to ID/AMS.

**Results:**

A total of 796 pharmacists with ID or AMS job responsibilities responded (3.5% response rate), with 607 working clinically or administratively in ID or AMS further categorized in four mutually exclusive groups based on formal and informal ID/AMS responsibilities. Respondents were predominantly female (66%), less than 40 years of age (59%) and white (82%). ID-specific training was completed by 41.8%, and 74% reported having student loan debt at graduation. Work-related activities were diverse and most frequently included: staffing or taking calls on weekends related to ID/AMS, AMS, educating learners or healthcare providers about ID-related topics, precepting learners, and conducting ID-related research and/or quality improvement projects. Respondents frequently indicated they lacked adequate job resources.

**Conclusions:**

The results highlight the extensive responsibilities placed on ID/AMS pharmacists to fulfill multiple roles. Pharmacists frequently lack ID-specific training or dedicated time for AMS responsibilities. The workforce is young, suggesting a need for both increased capacity for training programs and strategies for workforce retention.

## Introduction

Infectious diseases (ID) is an important practice specialty in many healthcare settings. Originally, ID pharmacists performed consultant roles to provide evidence-based pharmacotherapy recommendations.^1^ In 2007, U.S. guidelines recommended pharmacists with ID-specific training be core members of antimicrobial stewardship (AMS) programs.^2^ The Joint Commission has required AMS programs since 2017; however, the number of pharmacists with training specific to AMS or ID is inadequate.^3^ To date, there is no comprehensive survey describing the ID and AMS pharmacist workforce.^1,4^

This national survey aimed to understand the make-up of the workforce performing ID/AMS pharmacy tasks. Two objectives guided examination of survey responses: (1) describing demographic and work characteristics of the overall ID/AMS pharmacist workforce, and (2) examining differences in work activities, scope of practice, work settings, and challenges among ID/AMS pharmacists based on groupings according to whether ID and/or AMS responsibilities are part of formal versus informal job descriptions.

## Methods

### Survey creation

A group of ID pharmacists and members of the Midwest Pharmacy Workforce Consortium developed initial survey questions using the 2019 and 2022 National Pharmacy Workforce Study (NPWS) questionnaires as templates.^5,6^ An iterative process using *Qualtrics* ^*XM*^ survey platform was used to finalize questions, with feedback from members of the Society of Infectious Diseases Pharmacists (SIDP) and the American Society of HealthSystems Pharmacists (ASHP). Prior to distribution, the questionnaire was pilot-tested via a targeted sample of six ID/AMS pharmacists, leading to further refinement. The University of Minnesota Institutional Review Board considered the survey exempt from IRB review (STUDY00020263).

**Figure 1.**
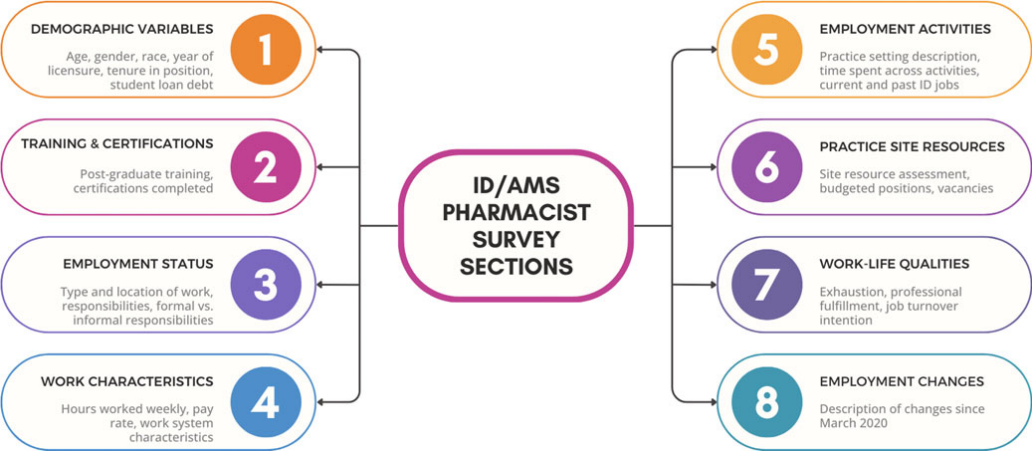
Description of the eight blocks of questions included in the infectious diseases (ID) and/or antimicrobial stewardship (AMS) pharmacist responsibilities survey. Blocks 1–6 are included in this manuscript, while 7–8 are reported in companion manuscripts.

Briefly, the survey consisted of eight sections: demographic variables, training and certifications, employment status, work characteristics, employment activities, practice site resources, work-life qualities, and employment changes (Figure 1). Demographic variables and training characteristics were derived from previous pharmacist workforce studies.^5–7^ Variable domain descriptions and an export of the full *Qualtrics^XM^* questions are provided in supplementary materials (Supplementary materials). Branching and skipping logic was used to direct respondents to relevant survey sections.

### Survey distribution and time line

The survey was distributed to 22,749 unique individuals via email to three overlapping listservs including: SIDP, the American College of Clinical Pharmacy, and a curated list of ASHP members who were either PGY2 residency program directors or people who purchased ID-related products or services from ASHP from 2020–2024. This large-scale distribution was intentional to ensure ID or AMS pharmacists of varied backgrounds were captured. The survey was open for four weeks, from 9/25/2024 to 10/24/2024.

### Identifying the sample of ID or AMS pharmacists

Pharmacists who responded to the following three questions were included: (1) employment status as working as a licensed pharmacist, (2) working in the U.S., and (3) engaged in ID or AMS activities. A respondent was considered engaged in ID or AMS activities if they reported involvement in at least one of 14 activities directly related to ID/AMS services (Supplementary Table 1).

### Respondent classifications by formal/informal ID and/or AMS responsibilities

We were interested in understanding differences between pharmacists engaged in ID and/or AMS responsibilities that were either formally or informally part of respondent job descriptions and, therefore, asked respondents to self-identify whether they had ID and/or AMS job responsibilities and whether these were formal or informal (See Q1–5 in Supplementary *Qualtrics^XM^* survey question export). Formal responsibilities were defined as those written into job descriptions, or within a memorandum of understanding or scope of work at their practice site. Definitions of ID or AMS job responsibilities were not provided in the survey. Response results revealed 80% of responding U.S. pharmacists practicing clinically could be classified into one of four self-categorized, mutually-exclusive groups based on the formal/informal nature of their reported ID and/or AMS responsibilities: (1) having formal ID and AMS responsibilities (“ID/AMS-Formal”), (2) having informal ID and AMS responsibilities (“ID/AMS-Informal”), (3) having formal or informal ID responsibilities exclusively (“ID-Exclusive”), (4) and having formal or informal AMS responsibilities exclusively (“AMS-Exclusive”) (Supplementary Table 2).

### Data analysis

Descriptive statistics and bivariate cross-tabulations were calculated for quantitative data and bivariate associations were tested using chi-squared tests. Differences in group means for continuous variables were tested using independent-samples t-tests or one-way analysis of variance (ANOVA). An *a priori* significance level of 0.05 was used, not adjusting for multiple comparisons because comparisons were considered hypothesis generating rather than confirming.^8^ Analyses were conducted using SPSS Statistics 27 (IBM Corp. Released 2020. IBM SPSS Statistics for Windows, Version 27.0. Armonk, NY: IBM Corp.). Responses to three open-ended questions were qualitatively analyzed (Supplementary materials).

## Results

### Characteristics of the entire ID/AMS pharmacist workforce sample

#### Demographic characteristics

A total of 796 pharmacists responded to the survey, for a response rate of 3.5%. Of these, 189 were excluded for various reasons (Supplementary materials), leaving a final sample of 607 ID/AMS pharmacists working clinically or administratively. Approximately two-thirds (66.7%) of respondents were female, and 59.0% were younger than 40 (Table 1). Respondents were mostly white (>80%) and over 95% were not of Hispanic, Latino, or Spanish origin. Most commonly, job responsibilities were formally included in practice site job descriptions.

**Table 1.** Demographic characteristics of pharmacists (n = 607) currently working clinically in the U.S. and who are involved in ID or AMS

Characteristic	Frequency, n (%^a^)
Age	
≤29	60 (11.2)
30–39	256 (47.8)
40–49	139 (25.9)
50–59	55 (10.3)
≥60	26 (4.9)
Gender	
Female	360 (66.4)
Male	181 (33.4)
Other	1 (0.2%)
Race	
White	433 (82.3)
Asian Indian	22 (4.2)
Chinese	18 (3.4)
More than 1 race	10 (1.9)
Black or African American	9 (1.7)
Filipino	8 (1.5)
Other Asian	7 (1.3)
Other	7 (1.3)
Vietnamese	6 (1.1)
Korean	3 (0.6)
American Indian or Alaskan Native	2 (0.4)
Japanese	1 (0.2)
Hispanic, Latino, or Spanish origin	
Not of Hispanic, Latino, or Spanish origin	499 (95.4)
Hispanic, Latino, or Spanish origin	24 (4.6)
U.S. region^a^	
South	194 (35.7)
Midwest	174 (32)
West	90 (16.6)
Northeast	85 (14)
Highest degree earned	
PharmD	505 (90.0)
MS or Doctoral Degree (other than PharmD)	56 (10.0)
Postgraduate residency or fellowship training^b^	
PGY1 residency	373 (61.4)
ID PGY2 residency	218 (35.9)
Non-ID PGY2 residency	41 (6.8)
ID fellowship	36 (5.9)
Both ID PGY2 and ID fellowship	13 (2.1)
No training beyond pharmacy school	20 (3.2)
Postgraduation certification^b^	
Any board certification	363 (59.8)
AMS certificate	173 (28.5)
Immunization certificate	234 (38.6)
Years in current position	
<5	248 (46.4)
5–9.99	147 (27.5)
10–19.99	103 (19.3)
20–29.99	26 (4.9)
30+	11 (2.1)
Student loan debt	
Had any amount of student loan debt at graduation	449 (74.0)
Student loan debt causes/caused feelings of distress (somewhat and strongly agree)	303/444 (68.2)
Student loan debt impacted career choice or past employment changes	191/446 (42.8)
Currently have student loan debt, if had student loan debt at graduation	212 (47.2)
Amount of student loan debt left to pay off	n = 211
>$200,000	44 (20.9)
$150,001–$200,000	34 (16.1)
$100,001–$150,000	49 (23.2)
$50,001–$100,000	34 (16.1)
$10,000–$50,000	42 (19.9)
<$10,000	8 (3.8)
ID responsibilities	
Exclusively formal	288 (47.4)
Exclusively informal	177 (29.2)
No ID responsibilities	106 (17.5)
Both formal and informal	36 (5.9)
AMS responsibilities	
Exclusively formal	318 (52.4)
Exclusively informal	150 (24.7)
No AMS responsibilities	94 (15.5)
Both formal and informal	45 (7.4)

a The percentage is a valid percentage excluding missing data.

b Category does not sum to 100%.

#### Postgraduate training

Postgraduate training was common: 66.4% reported completing either a PGY1only or both PGY1 and PGY2 residency programs (Table 1). ID-specific residency/fellowship training was less common (n = 241, 39.7%). Completing a PGY1 residency was more common among respondents <40 years old relative to older respondents (81.6% vs 50.5%; *P* < .001), as was completing an ID PGY2 residency (54.1% vs 19.5%; *P* < .001) (data by age not shown). Overall, 28.5% of pharmacists reported completing AMS certificate programs. More respondents aged 40 years or older, compared to younger respondents, reported AMS certification (42.3% vs 24.4%; *P* < .001) or having no postgraduate training or certification (5.9% vs 1.9%; *P* < .014). A total of 229 (37.7%) respondents reported holding current Board-Certified Infectious Diseases Pharmacist (BCIDP) certification.

#### Student loan debt

Seventy-four percent of respondents reported having student loan debt at graduation, with debt causing feelings of distress (68%) or impacting career choice or employment changes (42.4%) (Table 1). Of 219 respondents with debt remaining, the amount ranged from <$10,000 (3.8%) to >$200,000 (20.9%).

### Characteristics based on pharmacist groupings according to formal/informal and ID/AMS responsibilities

#### Pharmacist and work characteristics by ID and AMS job responsibility groups

Significant differences emerged across the four groups in terms of postgraduate training or certification and position tenure (Table 2). ID/AMS-Formal respondents completed PGY1, ID PGY2 residency, and were board certified in ID to a greater extent than others. Generally, a lower proportion of AMS-Exclusive pharmacists had completed different types of postgraduate training or certification. It was also more common for ID/AMS-Formal pharmacists to be in their current position for <5 years. Approximately three-fourths (74.4%) of ID/AMS-Formal pharmacists were younger than age 40 compared to half of ID/AMS-Informal or AMS-Exclusive pharmacists (*P* < .001).

**Table 2.** Respondent characteristics, current work position, setting, hours worked, and pay of pharmacists by ID/AMS formal/informal responsibility group (n = 484)

	ID/AMS-formal (n = 216)	ID/AMS-informal (n = 96)	ID-exclusive (n = 80)	AMS-exclusive (n = 92)
Postgraduate training/certifications				
PGY1 residency^a^	155 (71.8)^b^	54 (56.3)	43 (53.8)	42 (45.7)
ID PGY2 residency^a^	128 (59.3)	15 (15.6)	20 (25.0)	11 (12.0)
ID fellowship^a^	11 (5.1)	3 (3.1)	11 (13.8)	3 (3.3)
Board certified^a^	151 (69.9)	53 (55.2)	40 (50.0)	42 (45.7)
AMS certificate	52 (24.1)	23 (24.0)	19 (23.8)	31 (33.7)
Years in current position^a^				
<5	104 (53.9)	32 (40.5)	30 (44.1)	34 (40.0)
5–9.99	54 (28.0)	22 (27.8)	13 (19.1)	24 (28.2)
10–19.99	26 (13.5)	20 (25.3)	14 (20.6)	20 (23.5)
20–29.99	5 (2.6)	5 (6.3)	8 (11.8)	4 (4.7)
30+	4 (2.1)	0	3 (4.4)	3 (3.5)
Current position^c^				
Staff/clinical pharmacist^a^	181 (83.8)	83 (86.5)	58 (72.5)	69 (75.0)
System pharmacist/coordinator	44 (20.4)	12 (12.5)	6 (7.5)	15 (16.3)
Faculty member^a^	18 (8.8)	11 (11.5)	25 (31.3)	5 (5.4)
Consultant	7 (3.2)	4 (4.2)	3 (3.8)	3 (3.3)
Manager/assistant manager^a^	6 (2.8)	5 (5.2)	1 (1.3)	8 (8.7)
Chief pharmacy officer/director/assistant director^a^	2 (0.9)	3 (3.1)	1 (1.3)	7 (7.6)
Other	13 (6.0)	7 (7.3)	7 (8.8)	9 (9.8)
Primary place of employment^a^				
Hospital/health system inpatient pharmacy	147 (72.1)	68 (81.0)	40 (55.6)	72 (82.8)
Health system (supervision of multiple sites)	40 (19.6)	6 (7.1)	2 (2.8)	5 (5.7)
Academia	8 (3.9)	4 (4.8)	13 (18.1)	1 (1.1)
Outpatient clinic/ambulatory care	3 (1.5)	3 (3.6)	14 (19.4)	2 (2.3)
Other^d^	6 (2.8)	3 (3.5)	3 (3.8)	8 (8.7)
Hospital/health system characteristics	n = 187	n = 74	n = 42	n = 77
Small (<200 beds)^c^	45 (24.1)	17 (23.0)	8 (19.0)	29 (37.7)
Medium (200–500 beds)^c^	69 (36.9)	28 (37.8)	18 (42.9)	27 (35.1)
Large (>500 beds)^a^^,^^c^	70 (37.4)	18 (24.3)	16 (38.1)	14 (18.2)
Academic medical center^a^^,^^c^	87 (46.5)	31 (41.9)	20 (47.6)	14 (18.2)
Multi-hospital system^a^^,^^c^	61 (32.6)	16 (21.6)	9 (21.4)	14 (18.2)
Government hospital^c^	13 (7.0)	2 (2.7)	3 (7.1)	4 (5.2)
Hours worked				
Hours worked per week, mean (SD)	42.7 (6.4)	42.3 (7.6)	42.8 (10.1)	40.8 (6.3)
Hours worked per week, range	20–65	20–80	20–80	16–55
Working part-time (≤30 hours/week), n (%)	4 (1.9)	3 (3.1)	4 (5.0)	5 (5.4)
Allowed to work from home^a^	96 (44.4)	27 (28.1)	30 (37.5)	21 (22.8)
Hours worked from home, if allowed, mean (SD)	17.7 (11.3)	12.6 (10.7)	15.3 (11.0)	20.1 (14.1)
Hours worked from home, if allowed, range	1–40	2–40	2–40	4–40
How have weekly hours worked changed in the past year?	n = 193	n = 78	n = 66	n = 83
Stayed the same	145 (75.1)	55 (70.5)	52 (78.8)	63 (75.9)
Increased	34 (17.6)	18 (23.1)	12 (18.2)	15 (18.1)
Decreased	14 (7.3)	5 (6.4)	2 (3.0)	5 (6.0)
Pay				
Hourly wage	n = 31	n = 24	n = 23	n = 34
Mean (SD)	$78.67 (13.4)	$70.45 (14.1)	$75.49 (15.4)	$71.02 (10.0)
Range	$60.00–$112.00	$35.00–$93.00	$60.00–$112.32	$47.00–$100.50
Salary	n = 161	n = 55	n = 43	n = 50
Mean (SD)	$150,497 (25,846)	$145,196 (35,164)	$157,693 (27,804)	$141,539 (41,107)
Range	$16,969–$350,000	$50,000–$230,000	$120,000–$226,054	$30,000–$188,906

a *P* < .05, chi-squared test of independence across groups.

b All percentages are valid percentages excluding missing data.

c Percentages do not sum to 100% across categories.

d Other includes community pharmacy, health system outpatient pharmacy, home health/infusion, industry, mail-order pharmacy, managed care/pharmacy benefits manager, NH/long term care, professional trade association, specialty pharmacy and government.

Across groups, over 70% reported their role as a staff or clinical pharmacist (Table 2). Regarding primary place of employment, >80% of pharmacists in three of four groups reported working in a health-system, either an inpatient pharmacy or supervising multiple health-system sites. ID/AMS-Formal pharmacists more commonly supervised multiple sites within health-systems. Fewer AMS-Exclusive respondents (18.2%) reported working in academic medical centers.

Mean hours worked per week ranged from 40.8 (AMS-Exclusive) to 42.8 (ID-Exclusive), with no significant differences in hours worked. ID/AMS-Formal respondents (44.4%) were more frequently allowed to work from home versus other groups. Of 36% of respondents who reported working from home, a mean of 16.1 hours was worked from home per week. Approximately 20% of pharmacists across all four groups noted weekly work hours increased over the past year.

In terms of pay, 138 respondents reported being paid hourly with mean wage rates ranging from $70.45 to $78.67. For annual salaries, mean amounts ranged from $141,539 to $157,693. Neither hourly wage rate nor annual salary differed across groups.

#### Work responsibilities and time allocations

Pharmacists were most frequently engaged in these diverse activities across groups: staffing or taking weekend call related to ID/AMS (16.3%–100%), AMS (62.5%–98.6%), educating learners or healthcare providers about ID-related topics (72.8%–96.3%), precepting students/residents (75%–93.1%), and conducting ID-related research and/or quality improvement projects (47.8%–92.6%) (Table 3). A large proportion of pharmacists also had weekend staffing/call requirements unrelated to ID/AMS (up to 43.8% for ID/AMS-Informal). Across groups, 18.4% to 53.3% of respondents were managing patient medication therapy under collaborative practice agreements(CPAs), but only 7.1% to 15.3% had their employer billing for pharmacy services.

**Table 3.** Engagement in specific work responsibilities and typical time spent on work activities by ID/AMS formal/informal responsibility group (n = 484)

	ID/AMS-formal (n = 216)	ID/AMS-informal (n = 96)	ID-exclusive (n = 80)	AMS-exclusive (n = 92)
Activities for which the pharmacist is responsible, n (%)				
Staff or take calls on weekends related to ID/AMS^b^	216 (100)	21 (21.9)	15 (18.8)	15 (16.3)
Antimicrobial stewardship^b^	213 (98.6)	86 (89.6)	50 (62.5)	88 (95.7)
Educate learners/health providers on ID-related topics^b^	208 (96.3)	75 (78.1)	63 (78.8)	67 (72.8)
Precept students and/or residents^b^	201 (93.1)	81 (84.4)	66 (82.5)	69 (75.0)
Conduct ID-related research and/or QI projects^b^	200 (92.6)	56 (58.3)	54 (67.5)	44 (47.8)
Lead or contribute to therapy guidelines^b^	188 (87.0)	63 (65.6)	43 (53.8)	60 (65.2)
Lead/contribute to COVID-19 therapy guidelines^b^	148 (68.5)	37 (38.5)	22 (27.5)	33 (35.9)
Round with an ID consultant team^a^	135 (62.5)	26 (27.1)	21 (26.3)	13 (14.1)
Counsel patients^b^	76 (35.2)	46 (47.9)	42 (52.5)	27 (29.3)
Manage OPAT and/or CoPAT^b^	68 (31.5)	11 (11.5)	19 (23.8)	14 (15.2)
Staff or take calls on weekends NOT related to ID/AMS^b^	59 (27.3)	42 (43.8)	12 (15.0)	30 (32.6)
Manage HIV therapies^b^	55 (25.5)	15 (15.6)	18 (22.5)	4 (4.3)
Administer/prepare immunizations	16 (7.4)	12 (12.5)	9 (11.3)	12 (13.0)
Manage Hepatitis C therapy^b^	15 (6.9)	6 (6.3)	13 (16.3)	2 (2.2)
Identifying patients for and/or prescribing HIV PrEP^b^	14 (6.5)	3 (3.1)	16 (20.0)	4 (4.3)
ID point-of-care testing	5 (2.3)	0 (0)	1 (1.3)	2 (2.2)
Prescribing expedited partner therapy for STIs^b^	5 (2.3)	2 (2.1)	4 (5.0)	2 (2.2)
Percent of time performing activities in a typical week				
ID-related patient care services, mean (SD)^a^	37.3 (20.0)	20.2 (17.1)	24.7 (24.2)	23.6 (21.1)
Range	0–80	0–80	0–72	0–80
Number reporting 0%	8	8	19	10
Antibiotic use surveillance, mean (SD)^a^	21.9 (15.8)	13.9 (19.1)	8.2 (13.6)	15.5 (15.8)
Range	0–100	0–100	0–70	0–70
Number reporting 0%	10	23	40	13
ID-related education, mean (SD)^a^	13.7 (9.3)	9.5 (9.8)	13.8 (14.4)	6.9 (7.2)
Range	0–80	0–40	0–75	0–40
Number reporting 0%	8	19	12	23
ID-related business/organization management/administration, mean (SD)^a^	9.9 (11.5)	5.3 (7.0)	3.4 (5.5)	6.1 (11.6)
Range	0–65	0–30	0–30	0–80
Number reporting 0%	42	39	42	39
ID-related research/scholarship, mean (SD)^a^	6.2 (7.3)	3.6 (7.7)	11.0 (19.4)	2.4 (5.1)
Range	0–50	0–50	0–80	0–25
Number reporting 0%	65	49	33	60
Non-ID-related patient care services, mean (SD)^a^	4.6 (11.3)	30.8 (28.7)	16.6 (26.7)	25.4 (25.1)
Range	0–70	0–90	0–90	0–80
Number reporting 0%	137	24	41	27
Administrative, mean (SD)	2.1 (4.7)	3.5 (9.0)	3.5 (8.1)	4.1 (10.2)
Range	0–30	0–50	0–80	0–80
Number reporting 0%	153	59	52	56
HIV and/or Hepatitis related care, mean (SD)^a^	1.8 (4.2)	0.6 (1.8)	8.1 (18.8)	0.7 (4.4)
Range	0–30	0–10	0–80	0–40
Number reporting 0%	148	69	56	81
Other, mean (SD)^a^	2.4 (7.2)	12.5 (22.0)	10.7 (24.9)	15.3 (24.4)
Range	0–50	0–94.5	0–98	0–85
Number reporting 0%	168	46	50	52
Employer billing for pharmacy services^b^	13 (7.3)	5 (7.1)	9 (15.3)	6 (8.5)
Manage patient medication therapy under a collaborative practice agreement^b^	49 (25.8)	16 (21.6)	32 (53.3)	14 (18.4)

Note. HIV, human immunodeficiency virus; STI, sexually transmitted infection; PrEP, preexposure prophylaxis; QI, quality improvement; OPAT, outpatient parenteral antimicrobial therapy; CoPAT, complex outpatient parenteral antimicrobial therapy.

a *P* < .05, ANOVA test of mean differences across groups.

b *P* < .05, chi-squared test of independence across groups.

Respondents spent varied amounts of time performing activities during a typical week (Table 3). In terms of antibiotic use surveillance, the largest proportion of time was spent by ID/AMS-Formal pharmacists (21.9%) and the smallest proportion of time was spent by ID-Exclusive pharmacists (8.2%). For ID-related patient care services, the largest proportion of time was spent by ID/AMS-Formal pharmacists (37.3%) and the smallest proportion of time was spent by ID/AMS-Informal pharmacists (20.2%). Finally, for non-ID-related patient care services, the largest proportion of time was spent by ID/AMS-Informal pharmacists (30.8%) and the smallest proportion spent by ID/AMS Formal pharmacists (4.6%).

### Work setting characteristics

#### Presence of postgraduate training programs

There was variability across groups in terms of PGY2 ID residency programs at work sites; for example, 8.7% for AMS-Exclusive to 37.5% for ID-Exclusive (Table 4). ID/AMS-Formal, ID/AMS-Informal, and ID-Exclusive respondents reported an average of 1.2 ID PGY2 resident positions and AMS-exclusive respondents reported an average of 2 ID PGY2 resident positions. Previous five-year vacancy rates for ID PGY2 positions were low and similar across groups. Mean number of fellowship positions per fellowship site varied (1.8–8.0) but did not differ. When trainee positions were vacant, preceptors were regularly (mean = 32%) required to cover their activities. ID/AMS-Informal pharmacists reported significantly fewer mean hours per week of dedicated time for resident/fellow training.

**Table 4.** Characteristics of ID/AMS educational resources and ID pharmacist involvement in ID/AMS education by ID/AMS formal/informal responsibility group (n = 484)

	ID/AMS-formal (n = 216)	ID/AMS-informal (n = 96)	ID-exclusive (n = 80)	AMS-exclusive (n = 92)
PGY2 ID residency at setting, n (%)^a^	76 (35.2)	25 (26.0)	30 (37.5)	8 (8.7)
PGY2 ID residency is accredited by ASHP, n (%)	73 (96.1)	25 (100)	26 (86.7)	7 (87.5)
Number of resident positions^b^, mean (SD)	1.2 (0.38)	1.2 (0.37)	1.2 (0.43)	2.0 (2.4)
Range	1–2	1–2	1–2	1–8
Unfilled resident positions in the past 5 years, mean (SD)	0.15 (0.56)	0.28 (0.73)	0.4 (0.86)	0.13 (0.35)
Range	0–4	0–3	0–3	0–1
Had zero vacancies, n (%)	67 (88.2)	21 (84.0)	23 (76.7)	7 (87.5)
ID fellowship				
ID fellowship at setting, n (%)	21 (9.7)	6 (6.3)	10 (12.5)	3 (3.3)
Number of fellowship positions, mean (SD)	3.8 (3.0)	1.8 (1.7)	4.3 (3.2)	8.0 (2.8)
Range	1–11	1–5	2–10	6–10
PGY2 residency or fellowship	n = 82	n = 26	n = 33	n = 10
Residents or fellows have time dedicated to ID/AMS activities, n (%)	80 (97.6)	26 (100)	32 (97.0)	9 (90.0)
Hours per week of dedicated time for residents or fellows^b^, mean (SD)	22.3 (14.1)	11.1 (11.9)	19.8 (14.1)	13.6 (11.5)
Range	0.25–40	1–40	1–40	2–32
If resident or fellow positions are vacant, preceptor required to cover their activities, n (%)	32 (39.0)	7 (26.9)	8 (24.2)	2 (20.0)
Activities related to ID or AMS education				
Rotations precepted annually by learner group^c^	n = 196^d^	n = 65^d^	n = 60^d^	n = 64^d^
Pharmacy students, mean (SD)	3.23 (2.7)	5.12 (13.1)	5.86 (9.6)	5.13 (5.1)
Range	0.5–16	1–100	1–50	1–18
Precepted zero rotations^a^, n (%)	49 (25.0)	8 (12.3)	16 (26.7)	26 (40.6)
PGY1 residents, mean (SD)	3.13 (1.8)	3.16 (2.0)	3.10 (3.1)	3.69 (3.6)
Range	1–12	1–8	1–20	1–14
Precepted no rotations^a^, n (%)	29 (14.8)	21 (32.3)	21 (35.0)	32 (50.0)
PGY2 residents, mean (SD)	2.58 (1.7)	2.63 (2.4)	1.93 (1.9)	3.71 (4.9)
Range	1–10	1–10	1–10	1–15
Precepted zero rotations^a^, n (%)	85 (43.4)	38 (58.5)	32 (53.3)	50 (78.1)
Fellows, mean (SD)	2.0 (1.2)	2	4 (5.4)	4 (0)
Range	1–3	2–2	1–12	4–4
Precepted zero rotations, n (%)	192 (98.0)	64 (98.5)	56 (93.3)	62 (96.9)
Medical students, mean (SD)	3.09 (2.0)	5.08 (6.3)	5.06 (5.9)	2.2 (1.8)
Range	1–10	1–20	1–24	1–5
Precepted zero rotations, n (%)	143 (73.0)	53 (81.5)	44 (73.3)	59 (92.2)

a *P* < .05 chi-squared test of association.

b *P* < .05 ANOVA, differences in group means.

c The mean is a conditional mean, conditioned on participating in rotations (i.e., zero values are excluded).

d Number of respondents who reported spending a non-zero percent of time in ID-related education activities.

#### Precepting

The mean and range numbers of learners precepted annually varied markedly across groups. ID/AMS-Formal pharmacists, significantly more than others, spent any amount of time in educational/training activities (Table 4). ID/AMS-Formal pharmacists precepted the following number of learners per year: pharmacy students (mean = 3.23 ± 2.3), PGY1 residents (mean = 3.13 ± 1.8), PGY2 residents (mean = 2.58 ± 1.7), fellows (mean = 2.00 ± 1.2), and medical students (mean = 3.09 ± 2). Across the four groups, it was significantly more common for AMS-Exclusive respondents to precept no pharmacy students and no PGY1 and no PGY2 learners relative to the other groups.

#### ID and/or AMS pharmacist staffing

Across groups, budgeted pharmacist FTEs with formal responsibilities for ID and/or AMS ranged from mean of 1.7 (AMS-Exclusive) to 2.8 (ID-Exclusive) (Table 5), with ID/AMS-Formal and ID-Exclusive respondents reporting significantly more budgeted FTEs. A mean of 32% to 57.5% of respondents across groups reported adequate numbers of budgeted FTEs having formal ID and/or AMS responsibilities.

**Table 5.** ID and/or AMS pharmacists work setting resources and characteristics by ID/AMS formal/informal responsibility group (n = 484)

	ID/AMS-formal (n = 216)	ID/AMS-informal (n = 96)	ID-exclusive (n = 80)	AMS-exclusive (n = 92)
ID and/or AMS pharmacist staffing				
Number of budgeted pharmacist FTEs with formal responsibilities for ID and/or AMS, mean (SD)^a^	2.7 (2.4)	2.1 (2.3)	2.8 (2.7)	1.7 (2.3)
Range	0–12	0–11	0–14	0–14
Zero budgeted pharmacist FTEs with formal responsibilities for ID and/or AMS, n (%)^b^^,^^c^	3 (1.4)	18 (18.8)	5 (6.3)	15 (16.3)
Budgeted FTEs for ID/AMS are adequate, n (%)^b^^,^^c^^,^^d^	71 (43.3)	21 (42.0)	23 (57.5)	17 (32.1)
Number of pharmacists employed				
Number of pharmacists employed to fulfill ID/AMS responsibilities, mean (SD)	2.9 (2.6)	2.7 (2.9)	3.3 (3.1)	3.1 (3.5)
Range	0.2–15	0–15	0–14	0–15
Zero pharmacists employed to fulfill ID/AMS responsibilities, n (%)^b^^,^^c^	0	7 (11.1)	3 (6.1)	6 (9.1)
Actual number of pharmacists employed to fulfill ID/AMS responsibilities equals budgeted FTEs, n (%)^b^^,^^c^	131 (74.0)	43 (68.3)	34 (69.4)	34 (51.5)
All FTE ID/AMS positions filled, n (%)^b^^,^^c^^,^^e^	170 (95.5)	41 (82.0)	41 (83.7)	47 (83.9)
Other workplace resources				
I do not have adequate resources to do my job, n (%)^b^^,^^c^	79 (42.0)	46 (58.2)	18 (31.6)	42 (51.2)
Missing responses	18	23	23	10
Resources considered lacking, n (%)^c^	n = 79	n = 46	n = 18	n = 42
Health information technology	59 (74.7)	27 (58.7)	10 (55.6)	25 (59.5)
Organizational support	49 (62.0)	34 (73.9)	10 (55.6)	26 (61.9)
Additional pharmacists	37 (46.8)	24 (52.2)	9 (50.0)	24 (57.1)
Physical space	17 (21.5)	11 (23.9)	4 (22.2)	15 (35.7)
Technician personnel	9 (11.4)	10 (21.7)	5 (27.8)	7 (16.7)
Other	18 (22.8)	14 (30.4)	6 (33.3)	7 (16.7)

a *P* < .05 ANOVA, differences in group means.

b Percentages are valid percentages excluding missing data.

c *P* < .05 chi-squared test of association.

d The denominator is respondents who provided a value, including zero, for budgeted pharmacist FTE positions.

e The denominator is respondents who provided a non-zero value for budgeted FTE pharmacist positions.

The mean number of pharmacists employed to fill budgeted pharmacist FTEs for formal ID and AMS responsibilities ranged from 2.7 (ID/AMS-Informal) to 3.3 (ID-Exclusive) with no significant differences. A majority of respondents in each group reported that pharmacists employed to fill budgeted FTE pharmacist positions for formal ID/AMS responsibilities equaled the number of budgeted FTE positions.

The proportion of pharmacists reporting all budgeted FTE pharmacist positions with ID/AMS responsibilities were filled ranged from 82.0% (ID/AMS-Informal) to 95.5% (ID/AMS-Formal). Free-text responses for why FTE positions weren’t filled generally represented shared work system needs such as: people resigned, undesirable shift times, relocation, and pay too low (data not shown). When pharmacists were asked to comment why more FTEs were needed, numerous themes emerged including: high workload, outpatient parenteral antimicrobial therapy (OPAT) expansion, needing more ID/AMS services for teams without coverage, and expanding ID/AMS programs (Supplementary Table 3).

#### Work setting resources and characteristics

Respondents reporting they lacked adequate resources to do their job ranged from 31.6% (ID-Exclusive) to 58.2% (ID/AMS-Informal), with such reports being significantly more likely for ID/AMS-Informal and AMS-Exclusive pharmacists (Table 5). For respondents reporting inadequate resources, health information technology (65.4%) and organizational support (64.3%) were most frequently cited. Free-text responses identified work setting resource needs representing three themes: (1) time and structure of AMS programs to include learning, administration, and patient care, (2) having physician/provider support, and (3) technology issues (Supplementary Table 4).

## Discussion

The goal of this study was to characterize the U.S. ID/AMS pharmacist workforce. Pharmacists with ID/AMS responsibilities have varying levels of training and responsibilities; the survey enabled us to identify four main categories of ID/AMS pharmacists working clinically or administratively and to explore important facets of workforce development and demands. Overall, most respondents were female, young, and white, consistent with 2023 national estimates of pharmacists working in hospitals.^9^ These data generate several important areas for future research. First, younger pharmacist age is correlated with higher burnout and shorter job tenure;^10^ therefore, it will be important to determine how best to sustain the ID/AMS workforce and promote career longevity. Second, student debt at graduation was notable—about three-quarters had debt and ∼ 21% had >$200,000 in remaining debt, while almost half reported that this debt influenced career choices or past employment changes. Considerable debt burden is concerning and may impact future entrance to and exit from the ID/AMS workforce. Third, the ID/AMS pharmacist workforce is not aligned with racial and ethnic characteristics of the U.S. population, suggesting a need to diversify the ID/AMS pharmacist training pipeline.^11–13^ Fourth, most pharmacists across groups reported workloads were too high or FTE/unit ratios were perceived as too low, and many reported inadequate job resources. Future research should examine best practices related to needs for, and use of, human and nonhuman resources to meet or surpass antibiotic quality use metrics across AMS programs.

The ID/AMS pharmacist workforce represented in this study reported more postgraduate training compared to the broader pharmacist workforce in the 2024 NPWS, where 35.4% and 13.7% of respondents younger than 40 years completed PGY1 or PGY2 residencies, respectively.^14^ This compares to 81.6% and 54.1% for the same age group of ID/AMS respondents (data by age not shown). The type of postgraduate training in our results differed by age. Responding pharmacists >40 years old were more likely to hold AMS certification, while younger respondents were more likely to have completed PGY1 or ID PGY2 residencies. This might be explained by older respondents assuming ID/AMS responsibilities earlier in their careers when ID-specific residency and fellowship training was not as common. A previous survey reported that AMS pharmacists without ID-specific training frequently assume informal AMS roles/responsibilities, often in addition to other job duties, which may compromise the ability to dedicate time to AMS.^15^

ID-specific training rates among younger respondents were higher in our study than a 2022 national sample of pharmacists performing AMS activities where only 24% completed ID-specific PGY2 or fellowship training.^15^ This likely reflects differences in respondent job responsibilities, since our survey captured any pharmacist with ID- or AMS-related responsibilities, as opposed to pharmacists with solely AMS responsibilities.^7^ However, it is also possible that pharmacists with higher levels of training or those more fully engaged in ID/AMS were more likely to complete the survey. As the need for ID/AMS activities grows in the U.S., the capacity of both residency/fellowship and certificate programs should be monitored.

Cumulatively, most respondents (>80%) across the four groups tended to be a staff or clinical pharmacist and employed in a health system. A shift toward system roles among pharmacists conducting AMS complements a prior smaller study in which 46% of respondents had multiple sites covered by a single stewardship pharmacist, although these roles were not specifically described as system roles.^7^ Integrated health systems are expanding in the U.S. and, in 2018, it was estimated that approximately 75% of U.S. hospitals were affiliated with a health system.^16^ Centralized stewardship programs embedded within health systems have emerged with the purpose of improving antimicrobial use. Future research could examine differences between centralized and decentralized AMS programs in terms of pharmacist staffing, responsibilities, non-personnel resources, and antibiotic use outcomes.

We identified several key differences in work responsibilities across the four groups. First, ID/AMS-Formal respondents spent a significantly larger proportion of time performing ID/AMS-specific activities, such as ID-related patient care services and antibiotic use surveillance (Table 3). These results likely indicate more protected time for such activities. In contrast, ID/AMS-Informal pharmacists who do not have these activities formally established in job descriptions reported more time spent on non-ID-related patient care services when compared to ID/AMS-Formal pharmacists. Notably, weekend staffing or call unrelated to ID/AMS was significantly more common for ID/AMS-Informal when compared to ID/AMS-Formal pharmacists. There were also many free-response comments indicating split job responsibilities leading to challenges for ID/AMS-Informal pharmacists, with a need for more FTEs. Suggested actions to promote programmatic changes and prevent AMS pharmacist burnout should be followed, including ensuring that AMS job descriptions are accurate with clearly-outlined leadership responsibilities and allocated time for AMS activities beyond clinical work.^17^

An alternative explanation for ID/AMS-Formal respondents spending significantly more time performing ID/AMS-specific activities is that this group was more likely to have completed postgraduate residency training. That is, AMS pharmacists with ID-specific training tend to undertake intermediate/advanced AMS activities and more advanced interventions.^15^ Organizations employing pharmacists with training that facilitates engagement in more advanced ID/AMS activities may improve patient outcomes, increasing reimbursement and accreditation potential.^18^ Additional formal and protected roles and/or education and training opportunities in ID and AMS may allow pharmacists to spend more time executing higher-level interventions.^17^ Additionally, the discrepancy of services provided under CPAs versus those billed under CPAs demonstrates that there are opportunities to increase billing for such services/interventions.

Education and training of various learners, including non-pharmacist healthcare providers, was reported as a frequent responsibility and all groups precepted pharmacy students, PGY1 and PGY2 residents, fellows, and medical students; however, there was considerable education/training variation between groups. As the need for ID-trained pharmacists increases, attention must be focused on additional time spent by the ID/AMS pharmacist workforce to educate various learners. Our data suggest that it is important to develop strategies ensuring ID/AMS pharmacists, burdened with increasing demand for their skills and knowledge, have adequate time and energy to devote to educational activities.

## Limitations

Three limitations could bias the results or interpretations of the results. First, although this is the first comprehensive workforce survey of ID/AMS pharmacists in the U.S., the response rate was low but we were unable to account for undeliverable or unread invitation emails. Nonresponse bias is possible; however, the degree or direction of response bias is unknown because a population-level sample of ID/AMS pharmacists does not exist. Second, participant responses may not reflect consistency in question interpretation. For example, professional standards may define “stewardship,”^2,3^ but it cannot be verified that everyone adhered to consistent definitions of terms or concepts in the survey. Finally, detail provided in free-text responses varied considerably, so authors’ thematic interpretation of responses may not fully concord with a respondent’s intended meaning.

## Conclusion

A national study representing 607 ID/AMS responding pharmacists showed most of this workforce is female and younger than 40 years old. Pharmacists with formal ID/AMS job responsibilities had more frequently completed specialized PGY2 ID training and more frequently performed ID-related activities. Expansion of health systems and/or hospitals and growth in patient volume appears to be stressing the current ID/AMS workforce as many described inadequate job resources. Growth in FTE positions needs to be commensurate with expansion to ensure appropriate antibiotic use. As the demands for ID/AMS pharmacists grow, the capacity of training programs should be closely monitored, along with strategies for workforce retention, burnout prevention, and debt relief.

## Supporting information

10.1017/ash.2026.10312.sm001 Hirsch et al. supplementary material Hirsch et al. supplementary material

## Data Availability

The data underlying this article will be shared on reasonable requests made to the corresponding author.

## Supplementary material

The supplementary material for this article can be found at https://doi.org/10.1017/ash.2026.10312
