# Supplementary material for: A national survey of the infectious diseases and antimicrobial stewardship pharmacist workforce in the United States: work settings, characteristics, employment activities, resources and needs
Source: Antimicrob Steward Healthc Epidemiol. 2026 Mar 27;6(1):e76. doi: 10.1017/ash.2026.10312 (PMC13104570; doi:10.1017/ash.2026.10312)
Supplement: Hirsch et al. supplementary material [file S2732494X2610312Xsup001.docx]

**SUPPLEMENTARY MATERIALS**

**Methods**

*Variable Domains Used in Analyses*

A subset of survey items was used for the present analysis, representing survey domains 1-6 (**Figure 1**). In addition to items related to demographics (e.g., age, gender, race) (Domain 1), education/training characteristics (e.g., degrees earned, completed post-graduate training or certifications) (Domain 2), and employment status (e.g., currently working, retired, unemployed) (Domain 3), the analysis focused on work characteristics and activities (Domains 4-5) and practice site resources (Domain 6). Among work characteristics (Domain 4), respondents were asked if they had ID or AMS job responsibilities at their work setting and to report if their responsibilities in either area were formal or informal or not applicable (See Q1-5 in Qualtrics survey question export). Formal responsibilities were defined as those written into their job description, or using a memorandum of understanding or scope of work at their practice site. The question had five response categories and used a “check all that apply” format. From the responses to the question, we created a four-by-four table (**Supplementary Table 2**) based on the formal/informal nature of ID responsibilities and the formal/informal nature of AMS responsibilities. The frequency of responses within the sixteen mutually exclusive cells in the table were used to create the four categories used in the analysis.

Work activities (Domain 5) were assessed in two ways. First, respondents reported whether they were responsible for each of 17 work activities consistent with pharmacist responsibilities, 14 of which were considered specific to ID/AMS pharmacists (**Supplementary Table 1**). Second, respondents reported the percent of their typical work week that they spend in each of nine broader work activity categories **(Supplementary Table 1).** Brief descriptions of each broader work activity were given and the question format forced the total percentage across the nine categories to sum to 100%. Additionally, all respondents reported whether their primary employer was billing for pharmacy services and whether pharmacists were managing patient medication therapy under a collaborative practice agreement.

The questionnaire allowed respondents to report resources present and needed at the practice site in which they were working clinically (Domain 6). Such information included both the full-time equivalent (FTE) and actual number of pharmacists who have formal ID and/or AMS responsibilities, whether all of the FTE positions are filled, and adequacy of budgeted FTE pharmacist positions and reasons why budgeted FTE pharmacist positions need to increase. An additional set of questions addressed the status of resources to accomplish job objectives and those resources that are inadequate.

*Survey distribution and timeline*

The survey was distributed to 22,749 unique individuals via email to three overlapping listservs including: SIDP (n = 8,041), the American College of Clinical Pharmacy (n = 13,126), and a curated list of ASHP members (n = 2,553) who were either PGY2 residency program directors or people who purchased ID-related products or services from ASHP from 2020-2024. This large-scale distribution was intentional to ensure ID or AMS pharmacists of varied backgrounds were captured. The survey was open for four weeks. An initial invitation email was sent on 9/25/2024, with reminder emails sent 10/2/2024 and 10/15/2024. The survey closed 10/24/2024.

*Qualitative data analysis*

One author (DAM) extracted responses to each open-ended question from the database and allocated them into themes. Two authors (AMG, EBH) reviewed theme allocations and either confirmed or suggested changes. Disagreements were discussed and concordance was achieved. Once themes were confirmed, frequencies were calculated across distinct responses (**Supplementary Table 3 and Supplementary Table 4**).

**Results**

*Survey response numbers and exclusion reasons*

A total of 796 pharmacists responded to the survey (3.5% response rate), 21 of whom did not answer the employment status question. Of 775 respondents reporting employment status, 16 were NOT practicing as a pharmacist, were retired (n = 5), or were unemployed (n = 6), and were excluded from analysis. The remaining 748 respondents reported working as a pharmacist, with 704 working in the U.S. Of these respondents, 628 (89.2%) were engaging in ID or AMS activities, which comprised 607 (96.7%) practicing clinically or administratively and 21 (3.3%) working in the pharmaceutical or diagnostic industry. The final sample of 607 pharmacists working clinically or administratively as ID or AMS pharmacists was used for data analysis.

**Supplementary Table 1**. Respondents were asked to report their time spent in nine broader work activity categories. Seventeen pharmacist activities were assessed in the survey; a respondent was considered to be engaged in ID or AMS activities if they reported involvement in at least one of the 14 activities directly related to ID or AMS services.

| **Specific work activities for which respondents were responsible** |
| --- |
| 1. Round with an ID consult team |
| 1. Conduct antimicrobial stewardship activities |
| 1. Administer or prepare immunizations |
| 1. Conduct point-of-care testing for infectious diseases |
| 1. Manage HIV therapies |
| 1. Manage Hepatitis C therapy |
| 1. Manage OPAT and/or CoPAT |
| 1. Involved in prescribing expedited partner therapy for sexually transmitted infections |
| 1. Involved in identifying patients for and/or prescribing HIV pre-exposure prophylaxis (PrEP) |
| 1. Lead or contribute to COVID-19 therapy guidelines |
| 1. Lead or contribute to therapy guidelines |
| 1. Precept students and/or residents^a^ |
| 1. Counsel patients^a^ |
| 1. Educate learners or healthcare providers on infectious diseases-related topics |
| 1. Conduct infectious diseases-related research and/or QI projects |
| 1. Staff or take call on weekends related to ID/stewardship |
| 1. Staff or take call on weekends NOT related to ID/stewardship^a^ |
| **Percent of time spent in nine broader work activity categories** |
| 1. *Antibiotic use surveillance* (e.g., reviewing and reporting antibiotic use, reviewing and reporting drug-pathogen test results, developing and reviewing antibiotic use policies and procedures) |
| 1. *ID-related patient care services* (e.g., making antibiotic duration of therapy recommendations, making intravenous-to-oral conversions, making antibiotic use recommendations, conducting pharmacokinetic dosing and adjustments, other services designed for patient antibiotic use management) |
| 1. *ID-related education* (e.g., teaching, precepting, and mentoring of students/trainees/technicians) |
| 1. *ID-related business/organization/management/administration* (e.g., serving on committees, managing personnel, finances, and operations) |
| 1. *ID-related research and scholarship* (e.g., discovery, development, and evaluation of products, services, and/or ideas) |
| 1. *Non-ID-related patient care services* (e.g., assessing and evaluating patient medication-related needs, monitoring and adjusting patients’ treatments to attain desired outcomes, other services designed for patient care management) |
| 1. *Administrative tasks* (e.g., discharge paperwork, prior authorizations, communication regarding insurance) |
| 1. *HIV and/or hepatitis related care* (inpatient or outpatient) |
| 1. *Other activities* (not described above) |

HIV: human immunodeficiency virus; STI: sexually transmitted infection; PrEP: pre-exposure prophylaxis; QI: quality improvement; OPAT: outpatient parenteral antimicrobial therapy; CoPAT: complex outpatient parenteral antimicrobial therapy

^a^Activity not considered directly related to ID or AMS services

**Supplementary Table 2.**

Cross-tabulation of responses to question Q1-5, based upon self-identification for provision of informal or formal ID and/or AMS job responsibilities.

|  | **No formal or informal AMS services** | **Formal AMS services only** | **Informal AMS services only** | **Both formal and informal AMS services** | **Count of responses** |
| --- | --- | --- | --- | --- | --- |
| **No formal or informal ID services** | 14 | 49 | 34 | 9 | 106 |
| **Formal ID services only** | 56 | 216 | 16 | 0 | 288 |
| **Informal ID services only** | 20 | 52 | 96 | 9 | 177 |
| **Both formal and informal ID services** | 4 | 1 | 4 | 27 | 36 |
| **Count of responses** | 94 | 318 | 150 | 45 | 607 |

**Supplementary Table 3**. Frequency of themes related to reasons why additional pharmacist FTEs are needed for ID and/or AMS responsibilities stratified by ID or AMS job responsibility and formal/informal nature of job responsibilities.

| **ID/AMS-Formal** |
| --- |
| Workload too high or FTE/unit ratio perceived as too low (n = 51) |
| Expansion of OPAT services (n = 32) |
| Need to provide more ID/AMS services to teams without coverage (high need/demand) (n = 15) |
| ID/AMS program is expanding in organization (new initiatives) (n = 10) |
| Too much time spent on administrative time vs. patient care (n = 9) |
| Split job duties for pharmacists causes problems (n = 9) |
| AMS not being covered well enough (n = 8) |
| Support planned improvements in current ID/AMS program (n = 7) |
| Pharmacy department not managing ID/AMS staffing well (n = 6) |
| Expansion to outpatient (n = 6) |
| Expansion to pediatrics (n = 5) |
| Activities not allocated appropriately impacting quality of the program (n = 4) |
| Health System/Hospital expansion (n = 4) |
| Patient complexity/acuity increasing (n = 4) |
| Teaching/education duties expanding (n = 4) |
| Patient volume increasing (n = 3) |
| Expansion to HIV clinic (n = 2) |
| Expansion to transplant (n = 2) |
| Expansion to transitions of care (n = 2) |
| New external standards (n = 2) |
| Too much Pharmacist responsibility / not enough dedicated physician support (n = 2)  Safety concerns |
| **ID/AMS-Informal** |
| Split job duties for pharmacists cause problems (n = 16) |
| Need to provide more ID/AMS services to teams without coverage (high need/demand) (n = 6) |
| Workload too high or FTE/unit ratio perceived as too low (n = 6) |
| No FTEs budgeted for AMS and/or ID activities (n = 5) |
| Expansion of OPAT (n = 4) |
| Patient volume increasing (n = 4) |
| Too much time spent on administrative time vs. patient care (n = 3) |
| Pharmacy department not managing ID/AMS staffing well (n = 2) |
| Health System/Hospital expansion (n = 2) |
| General workload too high (n = 2) |
| IT support needed |
| Expansion to pediatrics |
| Need more informed leadership |
| Patient complexity/acuity increasing |
| **ID Exclusive** |
| Need to provide more ID/AMS services to teams without coverage (high need/demand) (n = 4) |
| Expansion of OPAT (n = 4) |
| Support planned improvements in current ID/AMS program (n = 4) |
| Pharmacy department not managing ID/AMS staffing well (n = 3) |
| Workload too high or FTE/unit ratio perceived as too low |
| General workload too high |
| New external standards |
| Expansion to HIV clinic |
| AMS not being covered |
| **AMS Exclusive** |
| Split job duties for pharmacists cause problems (n = 12) |
| Support planned improvements in current ID/AMS program (n = 10) |
| Workload too high or FTE/unit ratio perceived as too low (n = 6) |
| ID/AMS program is expanding in organization (new initiatives) (n = 5) |
| Teaching/education duties expanding (4) |
| General workload too high (n = 3) |
| Expansion of OPAT (n = 3) |
| No FTEs budgeted for AMS and/or ID activities (n = 3) |
| Resources (n = 2) |
| Pharmacy department not managing ID/AMS staffing well (n = 2) |
| Health System/Hospital expansion (n = 2) |
| Need to provide more ID/AMS services to teams without coverage (high need/demand) |
| Not enough support from other providers |

Full-time equivalent (FTE); HIV: human immunodeficiency virus; OPAT: outpatient parenteral antimicrobial therapy

Note: Themes extracted from verbatim text provided by respondents when describing other resource needs. The number of themes is greater than number of respondents due to multiple themes provided in responses.

**Supplementary Table 4**. Themes for other work setting resource needs, stratified by ID or AMS job responsibility and formal/informal nature of job responsibilities.

| **ID/AMS-Formal (n = 16)** |
| --- |
| Physician support/involvement (n = 6) |
| Literature/library access (n = 3) |
| Physician FTE devoted to stewardship |
| OPAT resources |
| OPAT admin |
| Funds to attend scientific meetings |
| Clinical support (MAs, RNs) |
| Larger microbiology lab |
| Microbiology expertise |
| More ID pharmacist FTEs |
| Statistical software support |
| Improved stewardship program resources (learning, admin, patient care) |
| **ID/AMS-Informal (n = 14)** |
| More Pharmacist FTEs (n = 6) |
| Access to literature/library (n = 2) |
| More technology in microbiology lab |
| Updated physical facilities |
| Motivated staff |
| Better staff culture |
| Improved ID program |
| Better HR policies |
| Data analyst support |
| Formal job title |
| More pharmacist expertise due to high patient acuity |
| ID Physician on site |
| Improved pharmacist and technician work ethic |
| Improved pharmacist and technician knowledge |
| Program support (More innovation and problem solving) |
| Physician support/involvement |
| Updated laptop computer technology |
| **ID-Exclusive (n = 6)** |
| Coordination between ID pharmacists |
| IT support |
| Pharmacy leadership support |
| OPAT admin support |
| Physician support/involvement |
| Formalized stewardship approach |
| **AMS-Exclusive (n = 6)** |
| Access to literature/library (n =3) |
| Policies and protocols for program |
| Standardized ID treatment pathways |
| Physician support/involvement |
| Microbiology lab not up to date |
| Informatics staffing |
| Microbiology PhD |
| Rotational stewardship duties developed |

Full-time equivalent (FTE); HIV: human immunodeficiency virus; HR: human resources; IT: information technology; MA: medical assistant; OPAT: outpatient parenteral antimicrobial therapy; RN: registered nurse

Note: Themes extracted from verbatim text provided by respondents when describing other resource needs. The number of themes is greater than number of respondents due to multiple themes provided in responses.

**ID Workforce Survey for Distribution**

Qualtrics Survey Flow and Questions

Block: Default Question Block (1 Question)

Standard: Block 1 - Screening/inclusion questions (2 Questions)

Branch: New Branch

If

If Q1-1. Please check the category that best matches your current employment status. Practicing as a pharmacist, clinically Is Selected

And Q1-2. Is your primary place of pharmacy practice / work based in the United States (including US... No Is Selected

Or Q1-1. Please check the category that best matches your current employment status. Practicing as a pharmacist, administratively Is Selected

And Q1-2. Is your primary place of pharmacy practice / work based in the United States (including US... No Is Selected

Or Q1-1. Please check the category that best matches your current employment status. Pharmacist in academia/educator Is Selected

And Q1-2. Is your primary place of pharmacy practice / work based in the United States (including US... No Is Selected

EndSurvey:

Branch: New Branch

If

If Q1-1. Please check the category that best matches your current employment status. Pharmacist in pharmaceutical or diagnostic industry Is Selected

Block: Block 1 - Screening/inclusion questions (3 Questions)

Block: Block 2 - Employment activities (18 Questions)

Block: Block 3 - Demographics (17 Questions)

Block: Block 4 - Training & Certifications (6 Questions)

Block: Block 7 - Employment status change (7 Questions)

Block: Block 8 - Current Work (6 Questions)

Block: Block 9 - Exhaustion (1 Question)

Block: Block 10 - Fulfillment (1 Question)

Block: Block 11 - Job Turnover Intention (3 Questions)

Block: Block 12 - general comments (1 Question)

EndSurvey:

Branch: New Branch

If

If Q1-1. Please check the category that best matches your current employment status. Employed in a health-related field or position but NOT practicing as a pharmacist Is Selected

Or Q1-1. Please check the category that best matches your current employment status. Employed in a career that is NOT health-related Is Selected

Or Q1-1. Please check the category that best matches your current employment status. Retired Is Selected

Or Q1-1. Please check the category that best matches your current employment status. Unemployed Is Selected

Standard: Block 2a - Previous employment activities (excluded) (13 Questions)

Standard: Block 3a - Demographics for the excluded (13 Questions)

EndSurvey:

Branch: New Branch

If

If Q1-1. Please check the category that best matches your current employment status. Practicing as a pharmacist, clinically Is Selected

And Q1-2. Is your primary place of pharmacy practice / work based in the United States (including US... Yes Is Selected

Or Q1-1. Please check the category that best matches your current employment status. Practicing as a pharmacist, administratively Is Selected

And Q1-2. Is your primary place of pharmacy practice / work based in the United States (including US... Yes Is Selected

Or Q1-1. Please check the category that best matches your current employment status. Pharmacist in academia/educator Is Selected

And Q1-2. Is your primary place of pharmacy practice / work based in the United States (including US... Yes Is Selected

Block: Block 1 - Screening/inclusion questions (3 Questions)

Block: Block 2 - Employment activities (18 Questions)

Block: Block 3 - Demographics (17 Questions)

Block: Block 5 - Practice site info (9 Questions)

Block: Block 4 - Training & Certifications (6 Questions)

Block: Block 7 - Employment status change (7 Questions)

Block: Block 8 - Current Work (6 Questions)

Block: Block 9 - Exhaustion (1 Question)

Block: Block 10 - Fulfillment (1 Question)

Block: Block 11 - Job Turnover Intention (3 Questions)

Block: Block 12 - general comments (1 Question)

EndSurvey:

| Page Break |  |
| --- | --- |

Start of Block: Default Question Block

Cover Letter - Intro
You are invited to participate in a research study to describe the characteristics, scope, and functions of the pharmacist workforce responsible for infectious disease (ID)-related tasks in the United States. This study is being conducted by Betsy Hirsch, PharmD, FCCP, FIDP, FIDSA (University of Minnesota), and David Mott, PhD, FAPhA (UW-Madison), and is co-sponsored by the Society of Infectious Diseases Pharmacists (SIDP) and American Society of Health-System Pharmacists (ASHP). 
 
Participation in this survey is voluntary. If you agree to participate in this study, you will complete an electronic, online survey that takes approximately **15 minutes** to complete. Participating in this study may not benefit you directly, but it will help investigators learn more about the pharmacist workforce conducting ID-related tasks which could lead to future improvements. Please click this link to complete the survey. You can skip any survey questions that you do not want to answer, but note that **you will not be able to navigate backwards in the survey questions** due to branching logic. Even if you start the survey, you are not required to complete it. You can stop at any time. The survey is anonymous, and no one will be able to link your answers back to you. Please do not include your name or other information that could be used to identify you in the survey responses. The survey will close October 23, 2024.
 
If you have any questions about participating, please contact Betsy Hirsch (ebhirsch@umn.edu) or David Mott (david.mott@wisc.edu).

End of Block: Default Question Block

Start of Block: Block 1 - Screening/inclusion questions

Q1-1 Please check the category that best matches your current employment status.

- Practicing as a pharmacist, clinically
- Practicing as a pharmacist, administratively
- Pharmacist in academia/educator
- Pharmacist in pharmaceutical or diagnostic industry
- Employed in a health-related field or position but NOT practicing as a pharmacist
- Employed in a career that is NOT health-related
- Retired
- Unemployed

Display this question:

If Please check the category that best matches your current employment status. = Practicing as a pharmacist, clinically

Or Please check the category that best matches your current employment status. = Practicing as a pharmacist, administratively

Or Please check the category that best matches your current employment status. = Pharmacist in academia/educator

Or Please check the category that best matches your current employment status. = Pharmacist in pharmaceutical or diagnostic industry

Q1-2 Is your primary place of pharmacy practice / work based in the United States (including US territories and DC)?

- Yes
- No

End of Block: Block 1 - Screening/inclusion questions

Start of Block: Block 1 - Screening/inclusion questions

Q1-3 Which of the following describes your current position? Select all that apply.

- Staff / Clinical Pharmacist
- System Pharmacist / Coordinator
- Chief Pharmacy Officer / Director / Assistant Director of Pharmacy
- Manager / Assistant Manager
- Pharmaceutical or Diagnostic Industry
- Faculty Member
- Consultant
- Other (please describe) __________________________________________________

Q1-4 Please check all activities in which you have responsibilities in your role as a pharmacist. Please select all that apply.

- Round with an ID consult team
- Conduct antimicrobial stewardship activities
- Administer or prepare immunizations
- Conduct point-of-care testing for infectious diseases
- Manage HIV therapies
- Manage Hepatitis C therapy
- Manage OPAT and/or CoPAT
- Involved in prescribing expedited partner therapy for sexually transmitted infections
- Involved in identifying patients for and/or prescribing HIV pre-exposure prophylaxis (PrEP)
- Lead or contribute to COVID-19 therapy guidelines
- Lead or contribute to therapy guidelines
- Precept students and/or residents
- Counsel patients
- Educate learners or healthcare providers on infectious diseases-related topics
- Conduct infectious diseases-related research and/or QI projects
- Staff or take call on weekends related to ID/stewardship
- Staff or take call on weekends NOT related to ID/stewardship activities
- None of the above

Q1-5 We are interested in whether your job responsibilities related to infectious diseases and/or antimicrobial stewardship are formal or informal. Formal responsibilities are defined as responsibilities written in your job description and/or MOU/scope of work at practice site(s). Please select all that apply.

- I have formal job responsibilities in infectious diseases
- I have informal job responsibilities in infectious diseases
- I have formal job responsibilities in antimicrobial stewardship
- I have informal job responsibilities in antimicrobial stewardship
- N/A

End of Block: Block 1 - Screening/inclusion questions

Start of Block: Block 2 - Employment activities

Q2-1 Please check the ONE item that best describes the setting or type of workplace for your primary place of employment.

- Community pharmacy (i.e. independently-owned, chain, mass merchandiser, grocery store)
- Health system outpatient pharmacy
- Outpatient clinic/ Ambulatory Care
- Hospital/Health System inpatient pharmacy
- Health system (supervision of multiple sites)
- Academia
- Home health/infusion
- Industry
- Mail-order pharmacy
- Managed care/pharmacy benefit manager
- Nursing home/long term care
- Professional/trade association
- Specialty pharmacy
- Telehealth or tele-ASP
- Other (please describe) __________________________________________________

Display this question:

If Please check the ONE item that best describes the setting or type of workplace for your primary p... = Health system outpatient pharmacy

Or Please check the ONE item that best describes the setting or type of workplace for your primary p... = Hospital/Health System inpatient pharmacy

Or Please check the ONE item that best describes the setting or type of workplace for your primary p... = Health system (supervision of multiple sites)

Q2-1 hospital site Please check all that apply to best describe the hospital site(s) where you work or are represented in your health system.

- Critical access / safety net hospital
- Small hospital (fewer than 200 beds)
- Medium hospital (200-500 beds)
- Large hospital (more than 500 beds)
- Academic medical center
- Government hospital (e.g. Veteran's Affairs)
- Multiple hospitals within the same health system
- Other (please describe) __________________________________________________

Q2-2 Have you ever held a clinical position (prior and/or current) in which you were engaged in ID- and/or AMS-related activities?

- Yes
- No

Display this question:

If Have you ever held a clinical position (prior and/or current) in which you were engaged in ID- an... = Yes

| 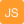 | 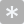 |
| --- | --- |

Q2-2 Yes How many prior ID- and/or AMS-related clinical positions (prior and/or current) have you held? (Enter the numeral)

________________________________________________________________

Display this question:

If Have you ever held a clinical position (prior and/or current) in which you were engaged in ID- an... = Yes

| 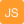 |
| --- |

Q2-2 Yes What was the total (i.e. cumulative) amount of time (in months or years) that you held any (prior and/or current) ID- and/or AMS-related clinical positions?

- Click to write Form Field 1 __________________________________________________
- Click to write Form Field 2 __________________________________________________

Display this question:

If Have you ever held a clinical position (prior and/or current) in which you were engaged in ID- an... = Yes

Q2-2 Yes In which practice settings did you hold the ID- and/or AMS-related clinical positions?

- Community pharmacy (i.e. independently-owned, chain, mass merchandiser, grocery store)
- Health System outpatient pharmacy
- Outpatient clinic/ Ambulatory Care
- Hospital/Health System- Inpatient
- Academia
- Home health/infusion
- Industry
- Mail-order pharmacy
- Managed care/pharmacy benefit manager
- Nursing home/long term care
- Professional/trade association
- Specialty pharmacy
- Other (please describe) __________________________________________________

Display this question:

If Have you ever held a clinical position (prior and/or current) in which you were engaged in ID- an... = Yes

Q2-2 Yes What reason(s) did you leave any previous ID- and/or AMS-related clinical positions?

- Family affairs
- High workload
- Inadequate pay
- Inadequate job satisfaction
- Personal health
- Other (please describe) __________________________________________________

Display this question:

If Have you ever held a clinical position (prior and/or current) in which you were engaged in ID- an... = No

Q2-2 No Is your current employment your first job?

- Yes
- No

| 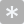 |
| --- |

Q2-3 Please indicate the average percent of time you dedicate to each of the following activities per week at your current primary employment (The total percentage cannot be greater than 100%).

⊗**Antibiotic Use Surveillance** (e.g., reviewing and reporting antibiotic use, reviewing and reporting drug-pathogen test results, developing and reviewing antibiotic use policies and procedures) : _______

⊗**ID-related Patient Care Services** (e.g., making antibiotic duration of therapy recommendations, making intravenous-to-oral conversions, making antibiotic use recommendations, conducting pharmacokinetic dosing and adjustments, making parenteral to oral conversions, other services designed for patient antibiotic use management) : _______

⊗**ID-related Education** (e.g., teaching, precepting, and mentoring of students/trainees/technicians) : _______

⊗**ID-related Business/Organization Management/Administration** (e.g., serving on committees, managing personnel, finances, and operations) : _______

⊗**ID-related Research/Scholarship** (e.g., discovery, development, and evaluation of products, services, and/or ideas) : _______

**Non ID-related Patient Care** (e.g., assessing and evaluating patient medication-related needs, monitoring and adjusting patients' treatments to attain desired outcomes, other services designed for patient care management) : _______

**Administrative Tasks** (e.g., discharge paperwork, prior authorizations, communication regarding insurance) : _______

**HIV and/or Hepatitis Related Care** (inpatient or outpatient) : _______

**Other Activities** (not described above) : _______

Total : ________

Display this question:

If Please indicate the average percent of time you dedicate to each of the following activities per... [ <strong>ID-related Education</strong> (e.g., teaching, precepting, and mentoring of students/trainees/technicians) ] > 0

Q2-3 Education prec What is the average number of rotations precepted each year for each type? (Assume one learner per rotation; if two learners precepted in a single rotation block, count this as two rotations.)

|  | Average number of rotations per year |
| --- | --- |
| Pharmacy Students |  |
| PGY1 Residents |  |
| PGY-2 Residents |  |
| Pharmacy fellows |  |
| Medical students, residents, or fellows |  |

Q2-4 Does your hospital/site have a PGY2 ID residency program (including accredited or non-accredited) or an ID fellowship program? Select all that apply.

- PGY2 ID residency
- ID fellowship program
- Neither

Display this question:

If Does your hospital/site have a PGY2 ID residency program (including accredited or non-accredited)... = PGY2 ID residency

| 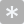 |
| --- |

Q2-5 How many PGY2 ID resident positions does your hospital/site have? (Enter the numeral)

________________________________________________________________

Display this question:

If Does your hospital/site have a PGY2 ID residency program (including accredited or non-accredited)... = PGY2 ID residency

| 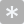 |
| --- |

Q2-6 Within the past 5 years, how many PGY2 ID positions have gone unfilled at your hospital? (Enter the numeral)

________________________________________________________________

Display this question:

If Does your hospital/site have a PGY2 ID residency program (including accredited or non-accredited)... = PGY2 ID residency

Q2-7 Is the PGY2 ID program accredited by ASHP?

- Yes
- No

Display this question:

If Does your hospital/site have a PGY2 ID residency program (including accredited or non-accredited)... = ID fellowship program

| 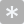 |
| --- |

Q2-8 How many ID fellowship positions does your hospital/site have? (Enter the numeral)

________________________________________________________________

Display this question:

If Does your hospital/site have a PGY2 ID residency program (including accredited or non-accredited)... = PGY2 ID residency

Or Does your hospital/site have a PGY2 ID residency program (including accredited or non-accredited)... = ID fellowship program

Q2-9 Do the PGY2 resident or fellow trainee(s) have independent, dedicated time towards antimicrobial stewardship or infectious diseases job responsibilities?

- Yes
- No

Display this question:

If Do the PGY2 resident or fellow trainee(s) have independent, dedicated time towards antimicrobial... = Yes

| 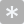 |
| --- |

Q2-10 How many hours per week (on average) does the PGY2 resident or fellow trainee(s) spend on these antimicrobial stewardship or infectious diseases job responsibilities?

________________________________________________________________

Display this question:

If Do the PGY2 resident or fellow trainee(s) have independent, dedicated time towards antimicrobial... = Yes

Q2-11 If the PGY2 or fellowship position is vacant, do you personally have to cover their job responsibilities?

- Yes
- No

End of Block: Block 2 - Employment activities

Start of Block: Block 3 - Demographics

| 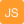 |
| --- |

Q3-1 What is your age in years?

________________________________________________________________

Q3-2 What year did you obtain your terminal pharmacy degree (PharmD, BSPharm)?

________________________________________________________________

Q3-3 How would you identify your gender?

- Male
- Female
- Non-binary
- Other __________________________________________________

Q3-4 What race(s) do you identify with? Please select all that apply.

- White
- Black or African American
- American Indian or Alaskan Native
- Chinese
- Filipino
- Asian Indian
- Other Asian
- Vietnamese
- Korean
- Japanese
- Native Hawaiian
- Samoan
- Chamorro
- Other Pacific Islander (please describe) __________________________________________________
- Other (please describe) __________________________________________________
- Prefer not to answer

Q3-5 Do you identify as being of Hispanic, Latino, or Spanish origin?

- No, not of Hispanic, Latino/Latina, or Spanish origin
- Yes, Mexican, Mexican Am, Chicano/Chicana
- Yes, Puerto Rican
- Yes, Cuban
- Yes, another Hispanic, Latino/Latina, or Spanish Origin (please describe) __________________________________________________
- Prefer not to answer

Q3-6 Do you personally identify as lesbian, gay, bisexual, or transgender?

- Yes
- No
- Prefer not to answer

Q3-7a In which state (or U.S. territory), is your primary place of employment located?

▼ Alabama ... I do not reside in the United States

Q3-7 Please enter the 5-digit ZIP code of your primary place of employment. If you have a split position, indicate the zip code for your clinical practice site.

________________________________________________________________

| 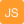 |
| --- |

Q3-8 How many years have you been in your current position?

________________________________________________________________

Q3-9 When you graduated from pharmacy school, did you have student loan debt?

- Yes
- No

Display this question:

If When you graduated from pharmacy school, did you have student loan debt? = Yes

Q3-9 Yes Do you currently have student loan debt remaining?

- Yes
- No

Display this question:

If Do you currently have student loan debt remaining? = Yes

Q3-9 Yes Yes What is the amount left to pay off?

- $10-50,000
- $50-100,000
- $100-150,000
- $150-200,000
- >$200,000

Display this question:

If When you graduated from pharmacy school, did you have student loan debt? = Yes

Q3-10 The student loan debt I have/had caused me distress.

- Strongly disagree
- Somewhat disagree
- Neither agree nor disagree
- Somewhat agree
- Strongly agree

Display this question:

If When you graduated from pharmacy school, did you have student loan debt? = Yes

Q3-11 Did your student loan debt impact your career choice or any past employment changes?

- Yes
- No

Display this question:

If Did your student loan debt impact your career choice or any past employment changes? = Yes

Q3-11Y If student loan debt impacted your career choice or past employment changes, please describe those here.

________________________________________________________________

________________________________________________________________

________________________________________________________________

________________________________________________________________

________________________________________________________________

Q3-12 Are you paid hourly or are you salaried?

- Hourly
- Salaried

| 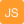 |
| --- |

Q3-13 Please report your current hourly rate or GROSS earnings per year for your **primary employment**.

- __________________________________________________
- __________________________________________________

End of Block: Block 3 - Demographics

Start of Block: Block 4 - Training & Certifications

Q4-1 Please report whether you completed each type of post-graduate training or each type of credential. Please select all that apply.

- PGY1 residency
- ID PGY2 residency
- PGY2 residency in non-ID specialty
- ID fellowship program
- Board certification
- Antimicrobial stewardship certificate program
- Immunization certification
- No post-graduate training or credential

Display this question:

If Please report whether you completed each type of post-graduate training or each type of credentia... = PGY2 residency in non-ID specialty

Q4-1 PGY2 in non-ID Which non-ID specialty PGY2(s) have you completed? Please select all that apply.

- Ambulatory care pharmacy
- Cardiology
- Community pharmacy
- Critical care
- Drug information
- Emergency medicine
- Health-system pharmacy administration
- HIV pharmacy
- Internal medicine
- Managed care pharmacy
- Medication-use safety
- Oncology
- Palliative care/pain management
- Pediatric pharmacy
- Pharmacotherapy
- Pharmacy informatics
- Psychiatric pharmacy
- Solid organ transplant

Display this question:

If Please report whether you completed each type of post-graduate training or each type of credentia... = Board certification

Q4-1 Yes board certi Which board certification(s) do you have? Please select all that apply.

- BCACP
- BCCCP
- BCCP
- BCEMP
- BCGP
- BCIDP
- BCNP
- BCNSP
- BCOP
- BCPP
- BCPPS
- BCPS
- BCSCP
- BCTXP
- AAHIVP/AAHIVE
- Other (please describe) __________________________________________________

Display this question:

If Please report whether you completed each type of post-graduate training or each type of credentia... = Antimicrobial stewardship certificate program

4-1 Yes stewardship Which stewardship certificate program(s) have you completed? Please select all that apply.

- SIDP
- MAD-ID
- SHEA
- IDSA
- Other (please describe) __________________________________________________

Q4-2 Please report what type of degree(s) you have earned. Please select all that apply.

- Bachelor's degree
- Master's degree
- PharmD
- Post-BS PharmD
- PhD
- Other (please describe) __________________________________________________

Display this question:

If Please report what type of degree(s) you have earned. Please select all that apply. = Master's degree

Q4-2 MS Which type of Master's degree(s) do you hold? Please select all that apply.

- MS
- MA
- MBA
- MHA
- MPH
- Other (please describe) __________________________________________________

End of Block: Block 4 - Training & Certifications

Start of Block: Block 7 - Employment status change

Q7-1 Between March 2020 (start of the COVID-19 pandemic) and today, did you experience any employment status change, defined as any change in your primary employer, remaining with your primary employer but changing a job position, dropping out of the workforce temporarily due to personal reasons and reentering the workforce, or dropping out of the workforce permanently due to personal reasons or retirement?

- Yes
- No

Display this question:

If Between March 2020 (start of the COVID-19 pandemic) and today, did you experience any employment... = Yes

Q7-1 Yes-1 How many employment status changes did you experience between March 2020 and today?

- 1
- 2
- more than 2

Display this question:

If Between March 2020 (start of the COVID-19 pandemic) and today, did you experience any employment... = Yes

Q7-1 Yes-2 Please report whether each of the following statements describes the type(s) of employment status change you experienced since March 2020.

Display this question:

If Between March 2020 (start of the COVID-19 pandemic) and today, did you experience any employment... = Yes

Q7-1 Yes-2-1 I left a clinical ID or ASP position and switched to a non-clinical ID or ASP position.

- Yes
- No

Display this question:

If Between March 2020 (start of the COVID-19 pandemic) and today, did you experience any employment... = Yes

Q7-1 if Yes 2-2 I left a clinical ID or ASP position and switched to a new clinical ID or ASP position.

- Yes
- No

Display this question:

If Between March 2020 (start of the COVID-19 pandemic) and today, did you experience any employment... = Yes

Q7-1 if Yes 2-3 Since March 2020, did you ever stop working altogether to take some time off from working? Please do not include personal COVID-related episodes of not working (i.e. quarantine, brief stoppages due to virus, etc.). Please check all that apply.

- Yes, I stopped voluntarily
- Yes, I stopped involuntarily
- No

Display this question:

If Since March 2020, did you ever stop working altogether to take some time off from working? Please... = Yes, I stopped voluntarily

Or Since March 2020, did you ever stop working altogether to take some time off from working? Please... = Yes, I stopped involuntarily

Q7-1 if Yes 2-4 Did you re-enter the workforce after taking time off?

- Yes
- No

End of Block: Block 7 - Employment status change

Start of Block: Block 8 - Current Work

| 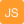 |
| --- |

Q8-1 How many hours per week do you work on average for your primary employer?

________________________________________________________________

Q8-2 During the past year, or at your most current primary employment, the number of actual hours you worked in a typical week for your primary employer has

- decreased
- stayed the same
- increased

| 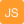 | 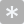 |
| --- | --- |

Q8-3 How many hours per week of a standard 40-hour work week does your employer allow you to work from home, on average?

________________________________________________________________

Q8-4 Please choose the most appropriate response for the following statement.

|  | Strongly disagree | Somewhat disagree | Neutral | Somewhat agree | Strongly agree |
| --- | --- | --- | --- | --- | --- |
| The work activities that I perform in my job extend beyond what I originally was hired to do. |  |  |  |  |  |
| I have a high level of autonomy in how I accomplish my work activities. |  |  |  |  |  |
| The number of pharmacists at my primary work setting is adequate to meet patient care needs or if in a system role, my organization's needs. |  |  |  |  |  |
| My organization implements strategies to improve well-being and resiliency for employees. |  |  |  |  |  |
| I often need to extend my workday (by spending time outside of my scheduled work hours) to accomplish everything for which I am responsible. |  |  |  |  |  |
| My manager/supervisor listens to me when I have concerns about my work. |  |  |  |  |  |

Q8-5 Do you manage patient medication therapy under a signed collaborative practice agreement?

- Yes
- No
- N/A

Q8-6 Is your employer billing for your pharmacy services?

- Yes
- No
- N/A

End of Block: Block 8 - Current Work

Start of Block: Block 9 - Exhaustion

Q9 Please indicate to what degree you have experienced the following at your primary employment.

|  | Not at all | Very little | Moderately | A lot | Totally |
| --- | --- | --- | --- | --- | --- |
| During the past 2 weeks I have felt a sense of dread when I think about work I have to do. |  |  |  |  |  |
| During the past 2 weeks I have felt physically exhausted at work. |  |  |  |  |  |
| During the past 2 weeks I have felt lacking in enthusiasm at work. |  |  |  |  |  |
| During the past 2 weeks I have felt emotionally exhausted at work. |  |  |  |  |  |

End of Block: Block 9 - Exhaustion

Start of Block: Block 10 - Fulfillment

Q10 Please indicate how true you feel the following statements are about yourself. At my primary employment, during the past two weeks:

|  | Not at all true | Somewhat true | Moderately true | Very true | Completely true |
| --- | --- | --- | --- | --- | --- |
| I feel happy at work. |  |  |  |  |  |
| I feel worthwhile at work. |  |  |  |  |  |
| My work is satisfying to me. |  |  |  |  |  |
| I feel in control when dealing with difficult problems at work. |  |  |  |  |  |
| My work is meaningful to me. |  |  |  |  |  |
| I’m contributing professionally in the ways I value most (e.g. patient care, teaching, research and leadership). |  |  |  |  |  |

End of Block: Block 10 - Fulfillment

Start of Block: Block 11 - Job Turnover Intention

Q11 Please indicate the likelihood of the following statement.

|  | Very unlikely | Unlikely | Neutral | Likely | Very likley |
| --- | --- | --- | --- | --- | --- |
| How likely is it that you will search for other employment within the next year? |  |  |  |  |  |
| How likely is it that you will actually leave your current employment within the next year? |  |  |  |  |  |
| How likely is it that you will be working as a pharmacist within the next year? |  |  |  |  |  |
| How likely is it that you will retire within the next year? |  |  |  |  |  |

Display this question:

If Please indicate the likelihood of the following statement. = Likely

Or Please indicate the likelihood of the following statement. = Very likley

Q11-1. If you are planning to leave your current employment within the next year, please explain why.

________________________________________________________________

________________________________________________________________

________________________________________________________________

________________________________________________________________

________________________________________________________________

Display this question:

If Please indicate the likelihood of the following statement. = Likely

Or Please indicate the likelihood of the following statement. = Very likley

Q11-2 If you are planning to leave your current employment within the next year, how important is work/life balance in your decision to leave your employer?

- Not important
- Somewhat important
- Very important

End of Block: Block 11 - Job Turnover Intention

Start of Block: Block 12 - general comments

Q12 Please provide any additional comments you would like us to know related to your role in infectious diseases and/or antibiotic stewardship

________________________________________________________________

________________________________________________________________

________________________________________________________________

________________________________________________________________

________________________________________________________________

End of Block: Block 12 - general comments

Start of Block: Block 2a - Previous employment activities (excluded)

Q1a-1 Were you working as a pharmacist as of March, 2020?

- Yes
- No

| 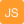 |
| --- |

Q1a-2 When did you stop working as a pharmacist?

|  |  |
| --- | --- |
| Month |  |
| Year |  |

Q2a-1 Please check the ONE item that best describes the setting or type of workplace for the most recent prior employment in a pharmacist role.

- Community pharmacy (i.e. independently-owned, chain, mass merchandiser, grocery store)
- Health system outpatient pharmacy
- Outpatient clinic/ Ambulatory Care
- Hospital/Health System inpatient phamracy
- Health system (supervision of multiple sites)
- Academia
- Home health/infusion
- Industry
- Mail-order pharmacy
- Managed care/pharmacy benefit manager
- Nursing home/long term care
- Professional/trade association
- Specialty pharmacy
- Other (please describe) __________________________________________________

Q2a-2 Did you ever hold a clinical position in which you were engaged in ID- and/or AMS-related activities?

- Yes
- No

Display this question:

If Did you ever hold a clinical position in which you were engaged in ID- and/or AMS-related activit... = Yes

| 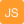 |
| --- |

Q2a-2 Yes-1 How many prior ID- and/or AMS-related clinical positions did you hold?

________________________________________________________________

Display this question:

If Did you ever hold a clinical position in which you were engaged in ID- and/or AMS-related activit... = Yes

| 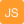 |
| --- |

Q2a-2 Yes-2 What was the total (i.e. cumulative) amount of time (in months or years) that you held any (prior) ID- and/or AMS-related clinical positions?

- Click to write Form Field 1 __________________________________________________
- Click to write Form Field 2 __________________________________________________

Display this question:

If Did you ever hold a clinical position in which you were engaged in ID- and/or AMS-related activit... = Yes

Q2a-2 Yes-3 In which practice settings did you hold the ID- and/or AMS-related clinical positions? Select all that apply.

- Community pharmacy (i.e. independently-owned, chain, mass merchandiser, grocery store)
- Health System outpatient pharmacy
- Outpatient clinic/ Ambulatory Care
- Hospital/Health System- Inpatient
- Academia
- Home health/infusion
- Industry
- Mail-order pharmacy
- Managed care/pharmacy benefit manager
- Nursing home/long term care
- Professional/trade association
- Specialty pharmacy
- Other (please describe) __________________________________________________

Display this question:

If Did you ever hold a clinical position in which you were engaged in ID- and/or AMS-related activit... = Yes

Q2a-2 Yes-4 Why did you leave any previous ID- and/or AMS-related clinical positions?

- Family affairs
- High workload
- Inadequate pay
- Inadequate job satisfaction
- Personal health
- Other (please describe) __________________________________________________

Display this question:

If Did you ever hold a clinical position in which you were engaged in ID- and/or AMS-related activit... = No

Q2a-2 No If you are still working, is this your first job?

- Yes
- No

Display this question:

If Did you ever hold a clinical position in which you were engaged in ID- and/or AMS-related activit... = Yes

Q1a-4 We are interested in whether your previous job responsibilities related to infectious diseases and/or antimicrobial stewardship were formal or informal, if there was any. Formal responsibilities are defined as responsibilities written in your job description. Please select all that apply.

- I had formal job responsibilities in infectious diseases
- I had informal job responsibilities in infectious diseases
- I had formal job responsibilities in antimicrobial stewardship
- I had informal job responsibilities in antimicrobial stewardship

Display this question:

If Did you ever hold a clinical position in which you were engaged in ID- and/or AMS-related activit... = Yes

Q1a-3 Please check all activities in which you had responsibilities in your most recent role in which you were engaged in ID- and/or AMS-related activities. Please select all that apply.

- Round with an ID consult team
- Conduct antimicrobial stewardship activities
- Administer or prepare immunizations
- Conduct point-of-care testing for infectious diseases
- Manage HIV therapies
- Manage Hepatitis C therapy
- Manage OPAT and/or CoPAT
- Involved in prescribing expedited partner therapy for sexually transmitted infections
- Involved in identifying patients for and/or prescribing HIV pre-exposure prophylaxis (PrEP)
- Lead or contribute to COVID-19 therapy guidelines
- Educate learners or healthcare providers on infectious diseases-related topics
- Conduct infectious diseases-related research and/or QI projects
- Staff or take call on weekends related to ID/stewardship
- Staff or take call on weekends NOT related to ID/stewardship

Display this question:

If Did you ever hold a clinical position in which you were engaged in ID- and/or AMS-related activit... = Yes

| 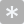 |
| --- |

Q2a-3 Please indicate the average percent of time you dedicated to each of the following activities per week at the most recent prior employment in which you were engaged in ID- and/or AMS-related activities (The total percentage should not be greater than 100%).

**Antibiotic Use Surveillance** (e.g., reviewing and reporting antibiotic use, reviewing and reporting drug-pathogen test results, developing and reviewing antibiotic use policies and procedures) : _______

**ID-related Patient Care Services** (e.g., making antibiotic duration of therapy recommendations, making intravenous-to-oral conversions, making antibiotic use recommendations, conducting pharmacokinetic dosing and adjustments, making parenteral to oral conversions, other services designed for patient antibiotic use management) : _______

**ID-related Education** (e.g. teaching, precepting, and mentoring of students/trainees/technicians) : _______

**ID-related Business/Organization Management/Administration** (e.g., serving on committees, managing personnel, finances, and operations) : _______

**ID-related Research/Scholarship** (e.g., discovery, development, and evaluation of products, services, and/or ideas) : _______

**Non-ID-related Clinical Patient Care**: (e.g. assessing and evaluating patient medication-related needs, monitoring and adjusting patients’ treatments to attain desired outcomes, and other services designed for patient care management) : _______

**Administrative Tasks** (e.g. discharge paperwork, prior authorizations, communication regarding insurance) : _______

**HIV and/or Hepatitis Related Care** (inpatient or outpatient) : _______

Total : ________

Display this question:

If Please indicate the average percent of time you dedicated to each of the following activities per... [ <strong>ID-related Education</strong> (e.g. teaching, precepting, and mentoring of students/trainees/technicians) ] > 0

Q2a-3 if precept What was the average number of rotations precepted each year for each type? (Assume one learner per rotation; if two learners precepted in a single rotation block, count this as two rotations.)

|  | Average number of rotations per year |
| --- | --- |
| Pharmacy Students |  |
| PGY1 Residents |  |
| PGY-2 Residents |  |
| Pharmacy fellows |  |
| Medical students/residents/fellows |  |

End of Block: Block 2a - Previous employment activities (excluded)

Start of Block: Block 3a - Demographics for the excluded

| 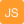 |
| --- |

3a-1 What is your age in years?

________________________________________________________________

3a-2 What year did you obtain your terminal pharmacy degree (PharmD, BSPharm)?

________________________________________________________________

3a-3 How would you identify your gender?

- Male
- Female
- Non-binary
- Other __________________________________________________

Q3a-4 What race(s) do you identify with? Please select all that apply.

- White
- Black or African American
- American Indian or Alaskan Native
- Chinese
- Filipino
- Asian Indian
- Other Asian
- Vietnamese
- Korean
- Japanese
- Native Hawaiian
- Samoan
- Chamorro
- Other Pacific Islander (please describe) __________________________________________________
- Other (please describe) __________________________________________________
- Prefer not to answer

Q3a-5 Do you identify as being of Hispanic, Latino, or Spanish origin?

- No, not of Hispanic, Latino/Latina, or Spanish origin
- Yes, Mexican, Mexican Am, Chicano/Chicana
- Yes, Puerto Rican
- Yes, Cuban
- Yes, another Hispanic, Latino/Latina, or Spanish Origin (please describe) __________________________________________________
- Prefer not to answer

3a-6 Do you personally identify as lesbian, gay, bisexual, or transgender?

- Yes
- No
- Prefer not to answer

3a-7 When you graduated from pharmacy school, did you have student loan debt?

- Yes
- No

Display this question:

If When you graduated from pharmacy school, did you have student loan debt? = Yes

3a-7 Yes Do you currently have student loan debt remaining?

- Yes
- No

Display this question:

If Do you currently have student loan debt remaining? = Yes

3a-7 Yes Yes What is the amount left to pay off?

- $10-50,000
- $50-100,000
- $100-150,000
- $150-200,000
- >$200,000

Display this question:

If When you graduated from pharmacy school, did you have student loan debt? = Yes

3a-8 The student loan debt I have/had caused me distress.

- Strongly disagree
- Somewhat disagree
- Neither agree nor disagree
- Somewhat agree
- Strongly agree

Display this question:

If When you graduated from pharmacy school, did you have student loan debt? = Yes

3a-9 Did your student loan debt impact your career choice or any past employment changes?

- Yes
- No

Display this question:

If Did your student loan debt impact your career choice or any past employment changes? = Yes

Q3a-10 If student loan debt impacted your career choice or past employment changes, please describe those here.

________________________________________________________________

________________________________________________________________

________________________________________________________________

________________________________________________________________

________________________________________________________________

| 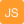 |
| --- |

Q3a-11 Please report your current hourly rate or GROSS earnings per year for your last primary employment role.

- __________________________________________________
- __________________________________________________

End of Block: Block 3a - Demographics for the excluded

Start of Block: Block 5 - Practice site info

Q5-1 screen Do you have a site in which you are currently practicing as a pharmacist?

- Yes
- No

Display this question:

If Do you have a site in which you are currently practicing as a pharmacist? = Yes

| 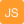 |
| --- |

Q5-1 At your primary practice site, how many FTE pharmacists have formal responsibilities related to infectious diseases and/or antibiotic stewardship?

- Please enter the number of FTE pharmacists __________________________________________________
- Don't know

Display this question:

If If At your primary practice site, how many FTE pharmacists have formal responsibilities related to i... Text Response Is Not Empty

Q5-1 Yes What is the actual number of pharmacists currently employed to fulfill these responsibilities?

- Please select to enter the number of pharmacists __________________________________________________
- Don't know

Display this question:

If Do you have a site in which you are currently practicing as a pharmacist? = Yes

| 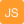 |
| --- |

Q5-2 Are all of the FTE positions with formal responsibilities related to infectious diseases or antibiotic stewardship filled at this time? Formal responsibilities are defined as responsibilities written in your job description.

- Yes
- No
- N/A

Display this question:

If Are all of the FTE positions with formal responsibilities related to infectious diseases or antib... = No

Q5-2 No If the FTE position is not filled, please describe why the position remains open.

________________________________________________________________

________________________________________________________________

________________________________________________________________

________________________________________________________________

________________________________________________________________

Display this question:

If Do you have a site in which you are currently practicing as a pharmacist? = Yes

| 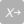 | 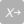 |
| --- | --- |

Q5-3 Is the budgeted FTE pharmacist staffing for infectious diseases and/or antibiotic stewardship activities adequate?

- Yes
- No
- Don't know
- N/A

Display this question:

If Is the budgeted FTE pharmacist staffing for infectious diseases and/or antibiotic stewardship act... = No

Q5-3 No Why does your practice site need additional budgeted FTE pharmacists for infectious diseases and/or antibiotic stewardship?

________________________________________________________________

________________________________________________________________

________________________________________________________________

________________________________________________________________

________________________________________________________________

Display this question:

If Do you have a site in which you are currently practicing as a pharmacist? = Yes

Q5-4 Do you feel like you have adequate resources to do your job?

- Yes
- No

Display this question:

If Do you feel like you have adequate resources to do your job? = No

Q5-4 No If you do not have adequate resources, which are you lacking?

- Health information technology
- Other pharmacist support (e.g., EM/critical care pharmacists)
- Technician personnel
- Physical space
- Organizational support
- Other (please describe) __________________________________________________

End of Block: Block 5 - Practice site info
